# M2 tumor-associated macrophages produce interleukin-17 to suppress oxaliplatin-induced apoptosis in hepatocellular carcinoma

**DOI:** 10.18632/oncotarget.17973

**Published:** 2017-05-18

**Authors:** Bin Guo, Leilei Li, Jiapei Guo, Aidong Liu, Jinghua Wu, Haixin Wang, Jun Shi, Dequan Pang, Qing Cao

**Affiliations:** ^1^ North China University of Science and Technology Affiliated Hospital, Tangshan, Hebei, China; ^2^ Hebei Medical University Second Hospital, Shijiazhuang, Hebei, China; ^3^ Hospital of Traditional Chinese Medicine of Tangshan City, Tangshan, Hebei, China

**Keywords:** hepatocellular carcinoma, M2 tumor-associated macrophages, interleukin-17, chaperone-mediated autophagy, cyclin D1

## Abstract

M2 macrophages are a major component of the tumor microenvironment and are important promoters of tumor occurrence and progression. In this study, we detected large numbers of M2 macrophages in hepatocellular carcinoma tissues using immunohistochemistry and immunofluorescence. Moreover, upon oxaliplatin treatment, the M2 macrophages overexpressed interleukin-17, an important inflammatory cytokine, and thus inhibited oxaliplatin-induced apoptosis. By knocking down the interleukin-17 receptor and lysosome-associated membrane protein 2A (a key protein in chaperone-mediated autophagy) in hepatocellular carcinoma cells, we found that interleukin-17 stimulated chaperone-mediated autophagy, which further suppressed apoptosis upon oxaliplatin treatment. Chaperone-mediated autophagy induced tolerance to oxaliplatin treatment by reducing cyclin D1 expression; thus, cyclin D1 overexpression stimulated oxaliplatin-induced apoptosis. In addition, cyclin D1 expression was inhibited by interleukin-17, but increased when the interleukin-17 receptor was knocked down. Thus M2 macrophages in the hepatocellular carcinoma microenvironment generate large amounts of interleukin-17, which suppress oxaliplatin-induced tumor cell apoptosis by activating chaperone-mediated autophagy and in turn reducing cyclin D1 expression. These findings may facilitate the development of novel therapeutic strategies for chemorefractory liver cancer.

## INTRODUCTION

Hepatocellular carcinoma (HCC) is a primary liver malignancy [1] and is one of the main causes of cancer-related death globally [2]. The survival rate of HCC patients is very low, and surgery is feasible for only a small percentage of patients. Therefore, chemotherapy is optimal for cases of inoperable HCC. However, chemoresistance and nonresponsiveness to HCC treatment are commonly observed. Studies on the chemoresistance or nonresponsiveness of tumor cells have mainly focused on genetic changes, drug efflux and metabolic drug deactivation [3]. However, it is now believed that the tumor microenvironment not only confers a poor prognosis, but also interferes with various tumor-directed therapies, including chemotherapy [4]. M2 tumor-associated macrophages (M2-TAMs), one type of TAM, are a major component of the tumor microenvironment, and have been shown to produce various cytokines that cause immunosuppression and resistance to chemotherapy [5, 6]. However, the mechanism of M2-TAM-induced chemoresistance remains unclear.

The tumor microenvironment is characterized by the accumulation of proinflammatory mediators and the infiltration of immune cells. Chronic inflammation has been shown to correlate significantly with tumor invasion, metastasis and chemoresistance [7]. Recently, it was reported that interleukin (IL)-17, a major proinflammatory cytokine in chronic inflammation, were significantly elevated in patients with HCC and promoted tumor growth and metastasis [8]. IL-17 is a pleiotropic cytokine that elicits a wide spectrum of physiological and pathological events, including cell proliferation, inflammation, and even autophagy [9].

Autophagy in response to chemotherapy may be a survival mechanism that promotes chemoresistance, so selective inhibition of autophagy regulators could improve the success of chemotherapeutic regimes [10, 11]. Chaperone-mediated autophagy (CMA) is a form of autophagy that prevents apoptosis [12], and can induce chemotherapy resistance by stimulating the degradation of abnormal proteins within cells and organelles and reducing the levels of reactive oxygen species [13]. In addition, CMA is an important regulator of cell cycle progression in tumors during chemotherapy [14]. The progression of the cell cycle also determines whether apoptosis will occur, and is an important factor in the occurrence, development and treatment of tumors. It has been confirmed that cell growth arrest at G0/G1 is a major mechanism of chemotherapy resistance [15, 16]. Inhibition of cyclin D1, a key promoter of cell cycle progression that binds to cyclin-dependent kinase (CDK) 4/6, induces the cell cycle arrest in G0/G1 phase [17].

Therefore, we assessed IL-17 production by M2-TAMs in the HCC microenvironment and its relationship to chemoresistance in HCC cells. We also investigated the involvement of CMA in the resistance of HCC cells to oxaliplatin-induced apoptosis.

## RESULTS

### M2-TAM distribution in HCC tissues

**Figure 1:**
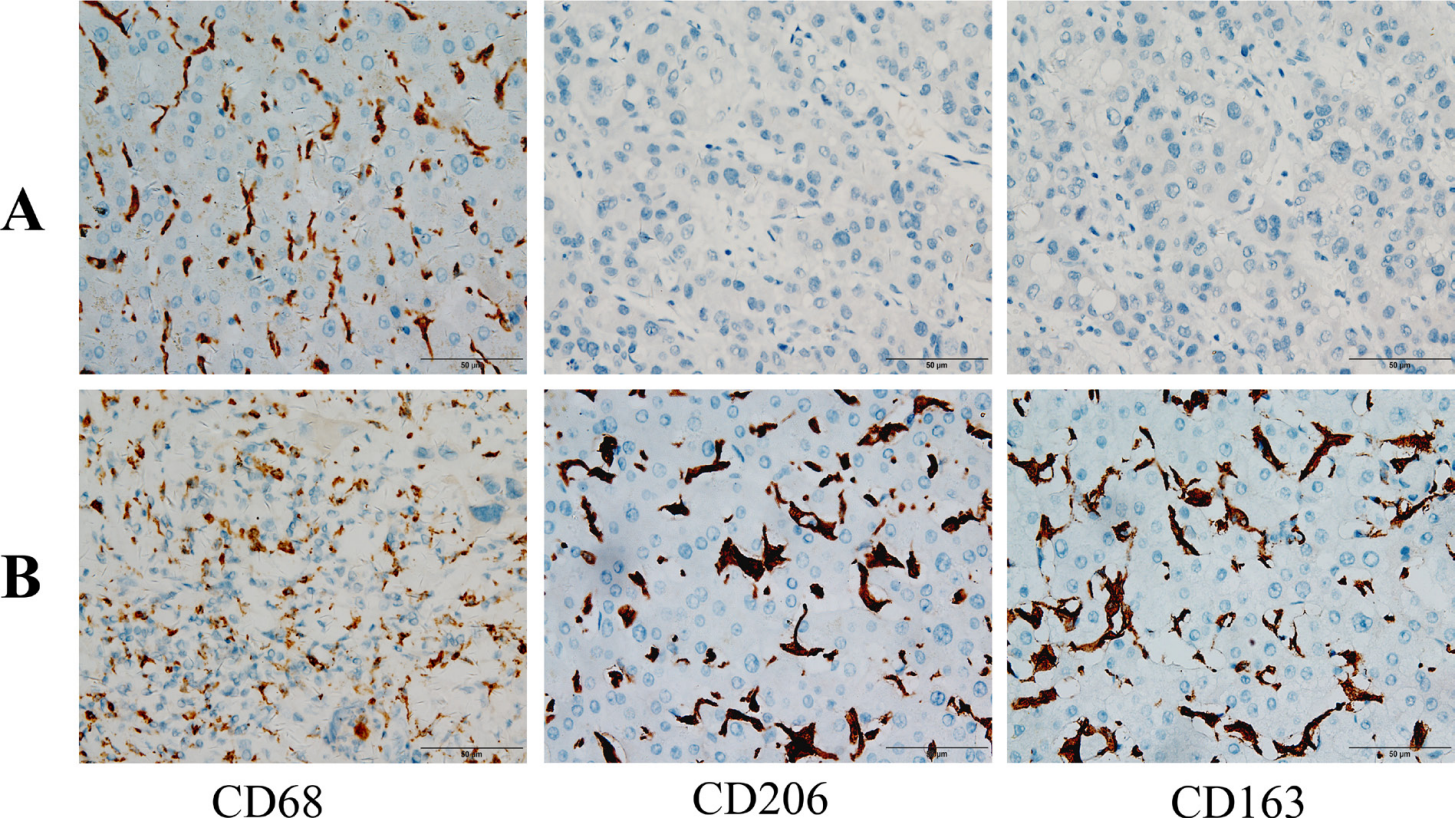
Immunohistochemical staining of HCC tissues (**A**) Normal hepatic tissues exhibiting CD68+, CD163+ and CD206+ cells (200×). (**B**) HCC tissues exhibiting CD68+, CD163+ and CD206+ cells (400×)

M2-TAMs comprise most of the cellular portion of the tumor microenvironment and are known to promote tumor progression and worsen the prognosis of tumor [18]. Therefore, we first used immunohistochemistry to detect the distribution of M2-TAMs in HCC tissues. As shown in Figure 1, the expression of CD68, CD163 and CD206, the M2-TAMs markers, were significantly higher in HCC tissues than in normal hepatic tissues (Figure 1).

**Figure 2:**
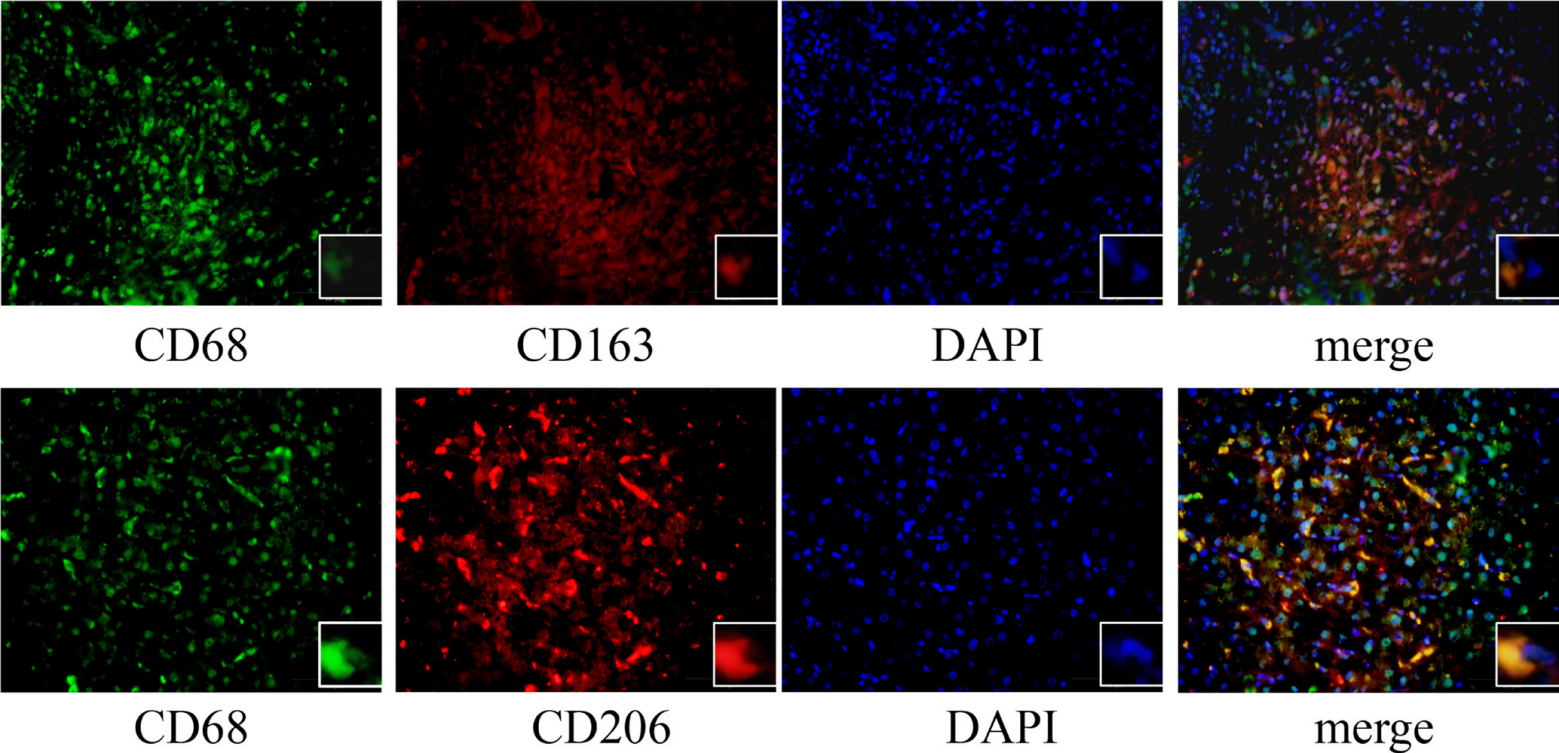
Immunofluorescence staining of HCC tissues The expression of CD68 (green) with CD163 (red) or CD206 (red) was detected with an immunofluorescence assay (400×).

We further defined the distribution of M2-TAMs in HCC tissues by performing an immunofluorescence assay. When HCC tissues were labeled with CD68 and CD163 or CD206, imaging revealed that CD68^+^ macrophages most heavily expressed CD163 or CD206 in HCC tissues (Figure 2). These results confirmed that a large number M2-TAMs were distributed in HCC tissues.

### IL-17 expression by M2-TAMs is augmented by oxaliplatin treatment

**Figure 3:**
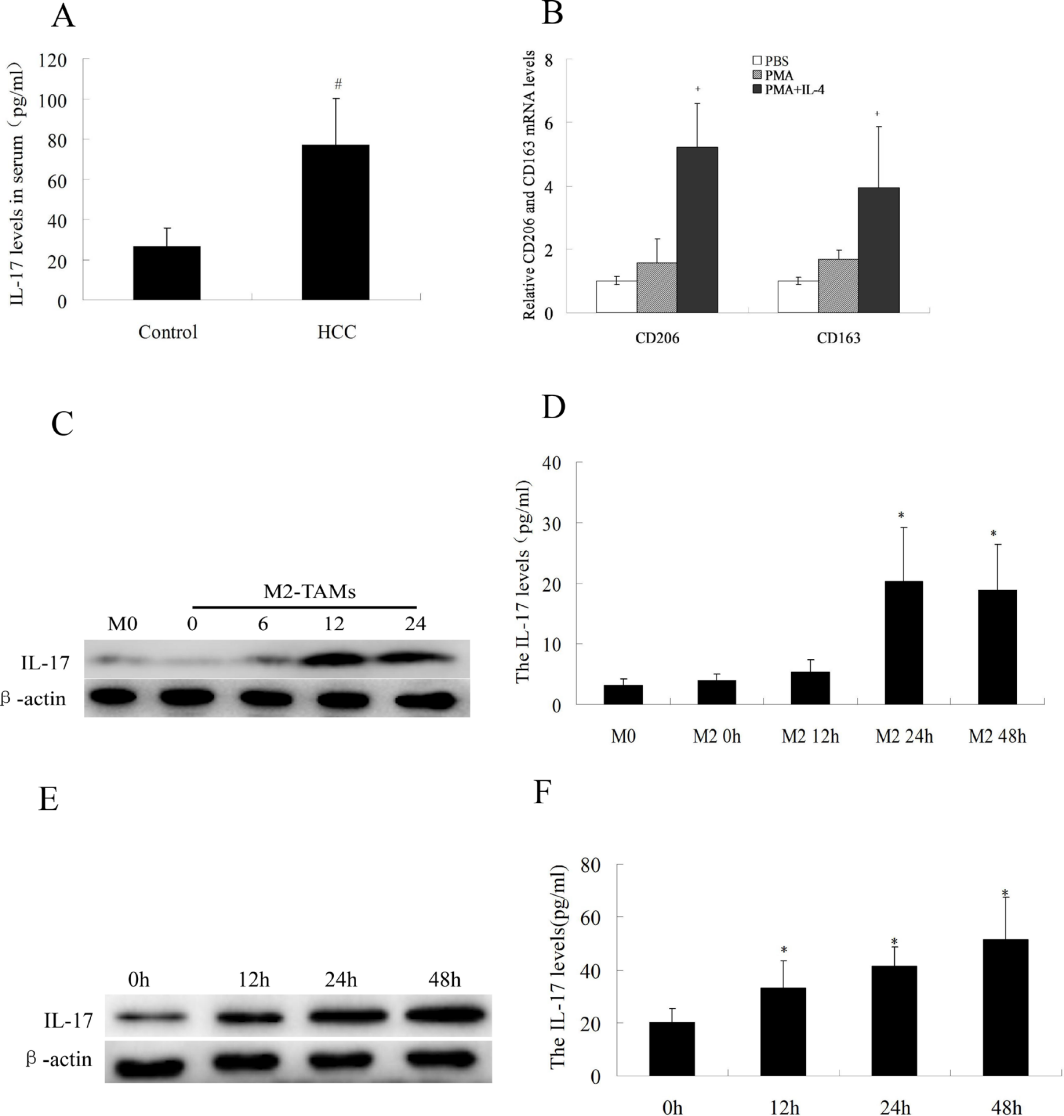
IL-17 levels in serum and HCC cells (**A**) IL-17 levels were analyzed by ELISA in serum samples from 30 HCC patients and 30 healthy controls. (**B**) After THP-1 cells were treated with 100 ng/mL PMA for 24 h and 100 ng/mL IL-4 for another 48 h, CD163 and CD206 were detected by quantitative real-time PCR. (**C**) IL-17 levels in the cytoplasm of M2-TAMs at various times were analyzed by Western blotting. (**D**) IL-17 levels in cell culture supernatants from M2-TAMs at various times were analyzed by ELISA. (**E**) IL-17 levels in the cytoplasm of M2-TAMs treated with 20 μg/mL oxaliplatin for various times were analyzed by Western blotting. (**F**) IL-17 levels in cell culture supernatants from M2-TAMs treated with 20 μg/mL oxaliplatin for various times were analyzed by ELISA. ^#^*p* < 0.05 vs. control group; *p < 0.05 vs. M0 cells (inactive macrophages); *^+^p* < 0.05 vs. PMA group.

Previous studies have shown that when HCC is associated with a chronic inflammatory state, it is often accompanied by cytokine abnormalities that promote tumor progression [19, 20]. Therefore, we measured the levels of IL-17, one of the inflammatory cytokines, in HCC patients. Serum IL-17 levels were significantly greater in HCC patients (77.36 ± 22.90 pg/mL) than in control subjects (26.65 ± 8.92 pg/mL) (Figure 3A).

It has been reported that IL-17 is secreted not only by TH17 cells, but also by thymocytes, endotheliocytes, epithelial cells, and even macrophages [21–22]. Considering the abundant distribution of M2-TAMs in HCC tissue and the elevated serum levels of IL-17 in HCC patients, we evaluated whether M2-TAMs expressed IL-17. First, we treated THP-1 cells with PMA and IL-4, and thus successfully differentiated them into M2-TAMs (Figure 3B). Then, we performed ELISA and Western blotting to determine the levels of IL-17 expressed by M2-TAMs. As shown in Figure 3C and 3D, at 24 h, IL-17 levels in the cytoplasm increased (as detected by Western blotting), and IL-17 levels in M2-TAM cell supernatants increased (as detected by ELISA). Moreover, the expression of IL-17 by M2-TAMs was obviously increased by oxaliplatin treatment (Figure 3E and 3F). These data suggested that M2-TAMs can produce IL-17, and that their IL-17 production is amplified by oxaliplatin treatment.

### IL-17 reduces oxaliplatin-induced apoptosis in HCC cells

**Figure 4:**
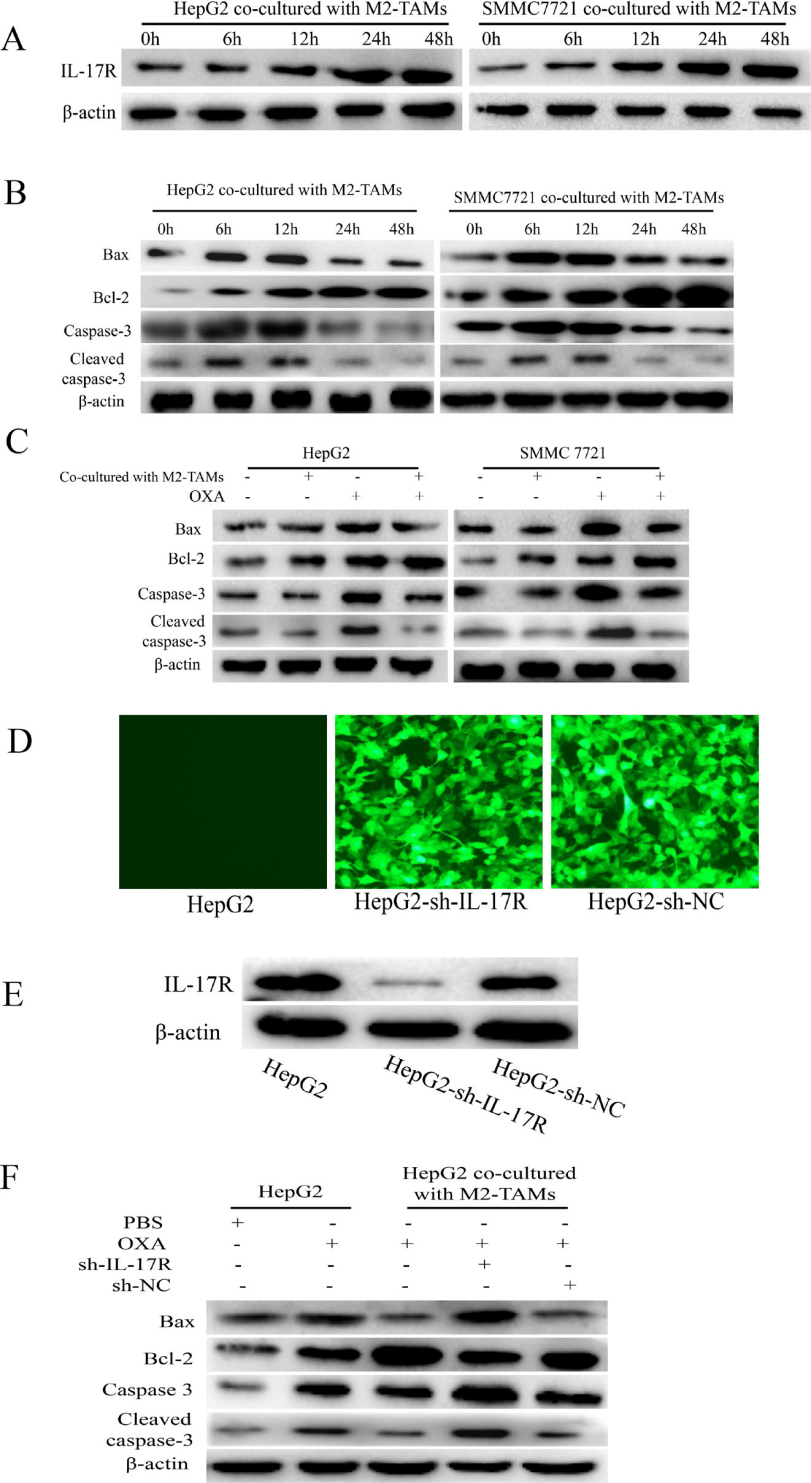
IL-17 reduces oxaliplatin-induced apoptosis in HCC cells (**A** and **B**) HepG2 and SMMC-7721 cells were co-cultured with M2-TAMs for 24 h and then treated with 20 μg/mL oxaliplatin for various times. IL-17R (A), BAX, BCL-2, caspase-3 and cleaved caspase-3 (B) were detected by Western blotting. (**C**) HepG2 and SMMC-7721 cells were cultured with or without M2-TAMs for 24 h and then treated with 20 μg/mL oxaliplatin for another 24 h. BAX, BCL-2, caspase-3 and cleaved caspase-3 were detected by Western blotting. (**D** and **E**) HepG2 cells were infected with lentiviruses expressing specific shRNAs (sh-IL-17R or sh-NC). The transfection efficiency was detected by fluorescence microscopy (200×) (D) and the knockdown efficiency was detected by Western blotting (E). (**F**) sh-IL-17R HepG2 cells were cultured with or without M2-TAMs for 24 h and then treated with 20 μg/mL oxaliplatin for another 24 h. BAX, BCL-2, caspase-3 and cleaved caspase-3 were detected by Western blotting.

The IL-17 receptor (IL-17R) on the cell surface, which specifically binds to IL-17, is expressed by many cell types, including macrophages, dendritic cells, epithelial cells and lung cells [23, 24]. Once IL-17 binds to IL-17R, the activated signaling pathway controls the relevant gene expression and cellular functions. To investigate whether M2-TAM-derived IL-17 was directed toward HCC cells, we co-cultured HCC cells with M2-TAMs, treated them with oxaliplatin for various times, and then detected the expression of IL-17R in the HCC cells by Western blotting. As shown in Figure 4A, IL-17R was expressed by HepG2 and SMMC-7721 cells co-cultured with M2-TAMs, and its expression gradually increased following oxaliplatin treatment.

These results led us to further examine the effects of elevated IL-17/IL-17R levels on HCC cell responsiveness to oxaliplatin treatment. We first detected the apoptosis of HepG2 and SMMC-7721 cells co-cultured with M2-TAMs and treated with oxaliplatin for various times. Western blotting revealed that BAX, caspase-3 and cleaved caspase-3 expression initially increased but then decreased, while BCL-2 expression gradually increased (Figure 4B). However, when HepG2 and SMMC-7721 cells were not co-incubated with M2-TAMs but were treated with oxaliplatin for 24 h, the levels of BAX, caspase-3 and cleaved caspase-3 clearly increased, while BCL-2 expression clearly decreased (Figure 4C). Therefore, it was evident that M2-TAMs suppressed oxaliplatin-induced HCC cell apoptosis.

Next, to determine whether IL-17 derived from M2-TAMs reduced oxaliplatin-induced HCC cell apoptosis, we used shRNAs to generate IL-17R knockdown cells (HepG2-sh-IL-17R) or control cells (HepG2-sh-NC) (Figure 4D and 4E). The cells were cultured with or without M2-TAMs for 24 h and then treated with 20 μg/mL oxaliplatin for another 24 h. BAX, caspase-3 and cleaved caspase-3 levels were markedly greater, while BCL-2 levels were lower in sh-IL-17R cells than in control cells (Figure 4F). These results demonstrated that IL-17 derived from M2-TAMs protects HCC cells from the apoptotic effects of oxaliplatin.

### IL-17 reduces HCC apoptosis by activating CMA

**Figure 5:**
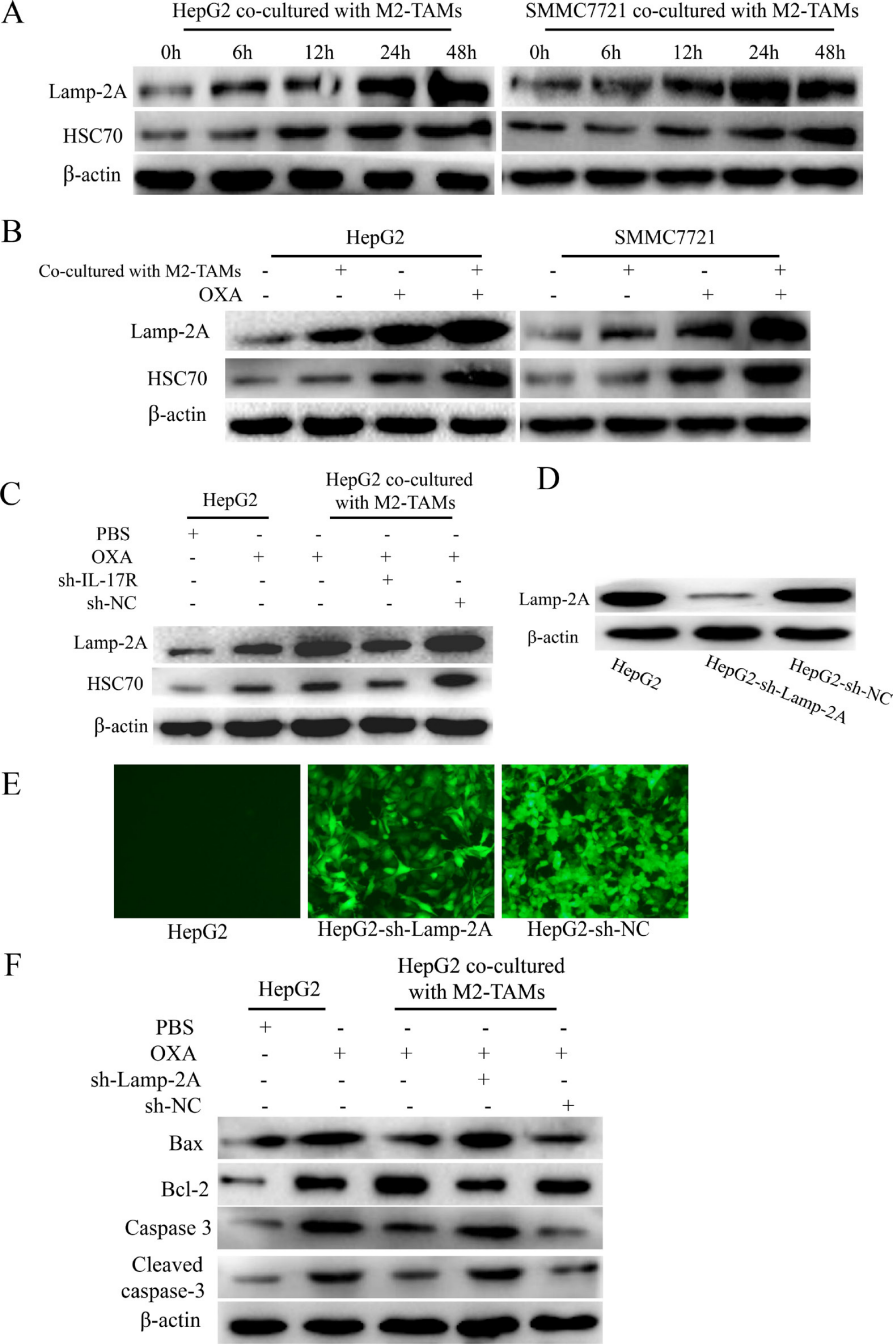
IL-17 reduces HCC cell apoptosis by activating CMA (**A**) HepG2 and SMMC-7721 cells were co-cultured with M2-TAMs for 24 h and then treated with 20 μg/mL oxaliplatin for various times. LAMP-2A and HSC70 levels were detected by Western blotting. (**B**) HepG2 and SMMC-7721 cells were cultured with or without M2-TAMs for 24 h and then treated with 20 μg/mL oxaliplatin for another 24 h. LAMP-2A and HSC70 levels were detected by Western blotting. (**C**) sh-IL-17R HepG2 cells were cultured with or without M2-TAMs for 24 h and then treated with 20 μg/mL oxaliplatin for another 24 h. LAMP-2A and HSC70 levels were detected by Western blotting. (**D** and **E**) HepG2 cells were infected with lentiviruses expressing a specific shRNA (sh-LAMP-2A). The transfection efficiency was detected by fluorescence microscopy (200×) (D) and the knockdown efficiency was detected by Western blotting (E). (**F**) sh-LAMP-2A HepG2 cells were cultured with or without M2-TAMs for 24 h and then treated with 20 μg/mL oxaliplatin for another 24 h. BAX, BCL-2, caspase-3 and cleaved caspase-3 were detected by Western blotting.

Recent studies have found that CMA is critical not only for the cellular response to stress, but also for cancer resistance to therapy [14]. To examine CMA activation upon oxaliplatin treatment of HCC cells, we detected the expression the LAMP-2A and HSC70 (key proteins indicating CMA activation [25]) in HCC cells co-cultured with M2-TAMs and treated with oxaliplatin for various times. As shown in Figure 5A, LAMP-2A and HSC70 expression increased gradually following oxaliplatin treatment, and peaked at 48 h.

Next, to test whether CMA activation was associated with the presence of M2-TAMs, we cultured HepG2 and SMMC-7721 cells with or without M2-TAMs for 24 h, and then treated them with oxaliplatin for another 24 h. LAMP-2A and HSC70 levels were obviously higher in HCC cells co-cultured with M2-TAMs than in those cultured without M2-TAMs (Figure 5B). In addition, sh-IL-17R HepG2 cells were cultured with or without M2-TAMs for 24 h and then treated with 20 μg/mL oxaliplatin for another 24 h. LAMP-2A and HSC70 levels were clearly lower in sh-IL-17R cells than in sh-NC cells or HepG2 cells co-cultured with M2-TAMs (Figure 5C). From this, we confirmed that IL-17 activates CMA in HCC cells upon oxaliplatin treatment.

Furthermore, to test whether CMA activation was responsible for suppressing oxaliplatin-induced apoptosis in HCC cells, we used shRNA to knock down LAMP-2A in HepG2 cells, cultured the cells with or without M2-TAMs for 24 h, and then treated them with 20 μg/mL oxaliplatin for another 24 h (Figure 5D and 5E). In HCC cells co-cultured with M2-TAMs, the extent of apoptosis was significantly greater in sh-LAMP-2A cells than in control cells (Figure 5F). Therefore, we confirmed that the activation of CMA by M2-TAM-derived IL-17 further suppresses oxaliplatin-induced apoptosis in HCC cells.

### Activation of CMA by IL-17 inhibits cyclin D1 expression and thus induces resistance to oxaliplatin

**Figure 6:**
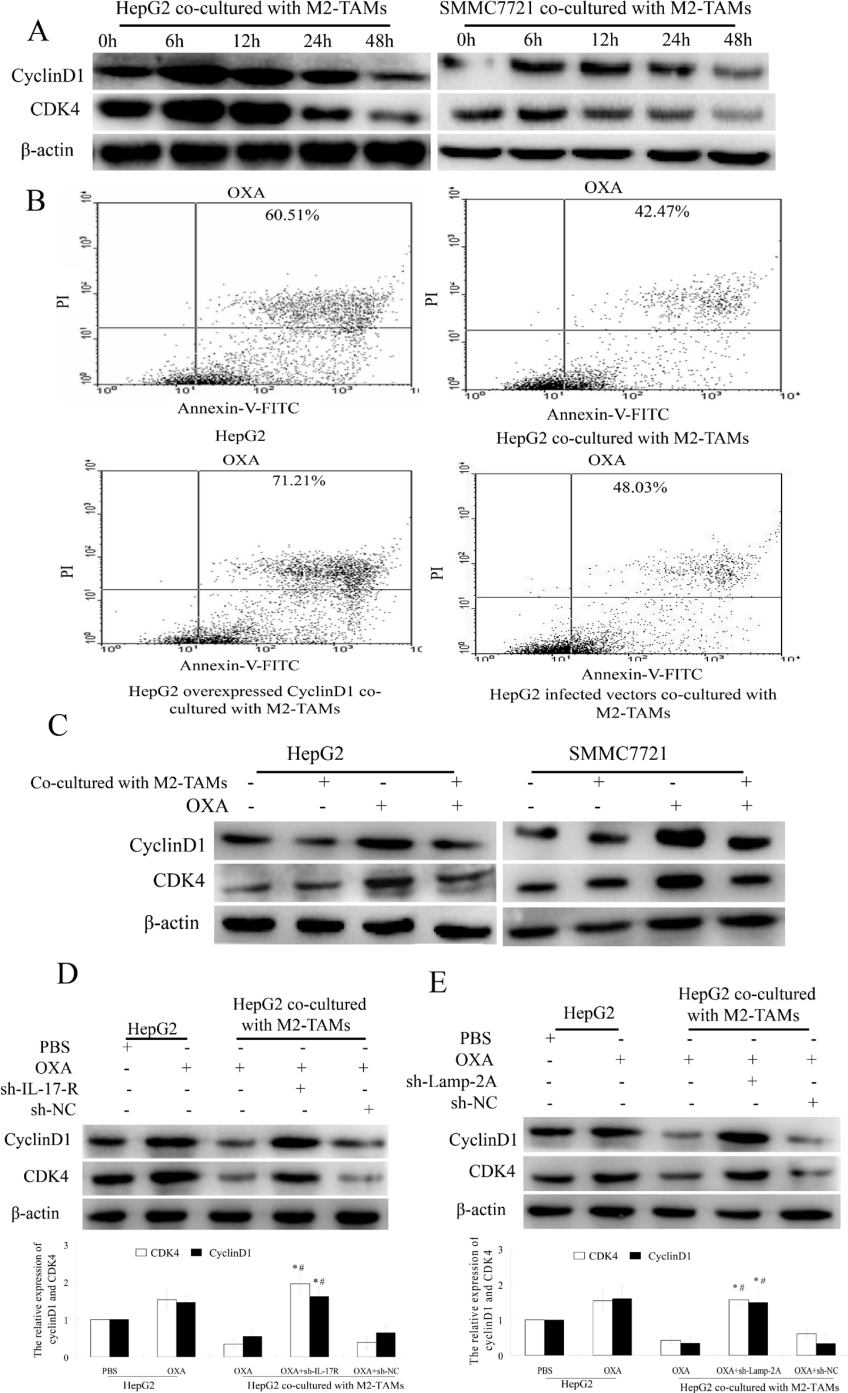
IL-17 reduces cyclin D1 expression by activating CMA (**A**) HepG2 and SMMC-7721 cells were co-cultured with M2-TAMs for 24 h and then treated with 20 μg/mL oxaliplatin for various times. Cyclin D1 and CDK4 were detected by Western blotting. (**B**) HepG2 cells were transfected with a cyclin D1 overexpression plasmid or a negative control vector and then co-cultured with M2-TAMs for 24 h. After oxaliplatin (20 μg/mL) treatment for another 24 h, apoptotic cells were detected by flow cytometry. (**C**) HepG2 and SMMC-7721 cells were cultured with or without M2-TAMs for 24 h and then treated with 20 μg/mL oxaliplatin for another 24 h. Cyclin D1 and CDK4 were detected by Western blotting. (**D**) sh-IL-17R HepG2 cells were cultured with or without M2-TAMs for 24 h and then treated with 20 μg/mL oxaliplatin for another 24 h. Cyclin D1 and CDK4 were detected by Western blotting. (**E**) sh-LAMP-2A HepG2 cells were cultured with or without M2-TAMs for 24 h and then treated with 20 μg/mL oxaliplatin for another 24 h. Cyclin D1 and CDK4 were detected by Western blotting.

Cyclin D1 forms a complex with CDK4, which phosphorylates Rb protein, thus inactivating Rb and its G1 phase-maintaining function, ultimately inducing the expression of proliferation-associated target genes [17]. The rate of cell cycle progression through G1 phase is mostly determined by the induction of cyclin D1. Some chemotherapeutic drugs such as oxaliplatin damage DNA and induce apoptosis without any apparent effects on cells in G0/G1 phase [26]. To determine whether cyclin D1 expression in HCC cells was reduced by M2-TAMs and whether this further inhibited apoptosis on oxaliplatin treatment, we first measured the changes in cyclin D1 and CDK4 expression in HCC cells co-cultured with M2-TAMs and treated with oxaliplatin for various times. Cyclin D1 and CDK4 levels began to decrease at 24 h and reached their lowest levels at 48 h (Figure 6A). Next, we evaluated the extent of oxaliplatin-induced apoptosis in HepG2 cells overexpressing the cyclin D1 gene and co-cultured with M2-TAMs. Upon oxaliplatin treatment, 71.21% of cyclin D1-overexpressing HepG2 cells were apoptotic, versus 42.47% of control HepG2 cells (Figure 6B). These results revealed that cyclin D1 stimulates oxaliplatin-induced apoptosis in HCC cells.

To determine whether the reduction of cyclin D1 was induced by M2-TAMs, we cultured HepG2 or SMMC-7721 cells with or without M2-TAMs and treated them with oxaliplatin for 24 h. As shown in Figure 6C, cyclin D1 and CDK4 levels were reduced when the cells were co-cultured with M2-TAMs. However, when the same conditions were tested in sh-IL-17R HepG2 cells, cyclin D1 and CDK4 levels increased (Figure 6D). These results revealed that cyclin D1 expression was reduced because M2-TAMs produced IL-17 in HCC cells upon oxaliplatin treatment.

Because IL-17 reduced HCC cell apoptosis mainly by activating CMA upon oxaliplatin treatment, we further tested whether CMA activation reduced HCC cell apoptosis by reducing cyclin D1 expression. We cultured sh-LAMP-2A HepG2 cells with or without M2-TAMs for 24 h, and then treated them with 20 μg/mL oxaliplatin for another 24 h. As shown in Figure 6E, cyclin D1 and CDK4 levels were higher in sh-LAMP-2A HepG2 cells than in control cells.

These results confirmed that IL-17 derived from M2-TAMs activates the CMA pathway, which reduces the expression of cyclin D1 and thus inhibits HCC apoptosis upon oxaliplatin treatment.

## DISCUSSION

Inducing cell death and inhibiting cell survival are the main principles of cancer chemotherapy. However, in HCC, resistance to chemotherapeutic agents is a major problem that limits the effectiveness of anticancer drugs. A variety of factors contribute to drug resistance, including host factors and specific genetic or epigenetic alternations in cancer cells [27]. However, recent studies have focused on how the tumor microenvironment promotes tumor cell invasion and resistance to chemotherapy [28, 29]. Macrophages are the main cell type in the tumor microenvironment, and possess molecular characteristics that permit neoplastic cells to escape immune surveillance [30–32]. The cytokines produced by macrophages in the tumor microenvironment promote cancer cell proliferation by enabling the cells to bypass apoptosis and even develop drug resistance [5, 6]. In this study, we found that M2-TAMs abundantly infiltrated HCC tissues, and that serum IL-17 levels were markedly elevated in HCC patients. The increase in IL-17 levels was mainly derived from M2-TAMs, and was augmented upon oxaliplatin treatment. Moreover, the increased levels of IL-17 contributed to the inhibition of apoptosis upon oxaliplatin treatment.

CMA is activated as a protective mechanism during chemotherapy, and thus causes the acquired resistance phenotype of some cancer cells [12, 33]. Substances secreted by M2-TAMs have been reported to activate autophagy and promote cancer progression and treatment resistance [34]. Here, we found that M2-TAMs activated CMA, which further reduced apoptosis following oxaliplatin treatment. As an important inflammatory cytokine secreted by M2-TAMs, IL-17 has enormous significance in inhibiting HCC cell apoptosis. We also found that IL-17 activated CMA, confirming that the secretion of IL-17 by M2-TAMs augmented oxaliplatin resistance by stimulating CMA.

Cyclin D1 and CDK4 are key cell cycle proteins [35], and have been found to be overexpressed in several types of human cancer [36, 37]. In addition, the attenuation of cell cycle progression reduces the susceptibility of tumor cells to chemotherapy and radiation in human carcinomas [38]. Wu et al. found that tolvaptan reduced the levels of cell cycle proteins and the associated kinases, thus delaying the cell cycle and reducing drug sensitivity in HepG2 cells [39]. In this study, we found that cyclin D1 expression was gradually reduced following oxaliplatin treatment, which thus inhibited apoptosis, whereas cyclin D1-overexpressing cells underwent a greater extent of apoptosis following oxaliplatin treatment.

It has long been thought that CDK and cyclin proteins are degraded through the ubiquitin pathway, but recently, the regulation of the cell cycle and the maintenance of cell renewal by CMA has become an important new research field [40]. In this study, the increase in cyclin D1 and CDK4 expression upon knockdown of LAMP-2A in HCC cells confirmed that CMA activation has the major effect of reducing cyclin D1 expression. Moreover, cyclin D1 expression was also reduced by IL-17 derived from M2-TAMs, but increased when IL-17R was knocked down in HCC cells.

In conclusion, our results confirmed that IL-17 was generated in large amounts by M2-TAMs distributed in the HCC microenvironment, which suppressed oxaliplatin-induced apoptosis in HCC cells by activating CMA and thus reducing cyclin D1 expression. These findings may facilitate the development of novel therapeutic strategies for chemorefractory liver cancer.

## MATERIALS AND METHODS

### Patient samples and tissue processing

We obtained a series of HCC specimens from 30 patients with pathologically confirmed HCC at North China University of Science and Technology Affiliated Hospital. No patient had received adjuvant chemotherapy, radiotherapy or surgery. Matched normal hepatic tissues were obtained from the Department of Anatomy. Control sera were collected from healthy subjects. All patients provided Institutional Review Board-approved informed consent before specimen collection.

### Reagents and antibodies

Antibodies against IL-17, IL-17 receptor (IL-17R), caspase-3, cleaved caspase-3, BAX, BCL-2, cyclin D1, CDK4, lysosome-associated membrane protein 2A (LAMP-2A), HSC70 and β-actin were obtained from Abcam (Cambridge, UK). Other reagents included anti-CD68 (Santa Cruz Biotechnology, Santa Cruz, CA, USA), anti-CD163 (Santa Cruz Biotechnology), anti-CD206 (Santa Cruz Biotechnology), tetramethylrhodamine isothiocyanate (TRITC) goat anti-mouse IgG (Boster, Wuhan, China) and fluorescein isothiocyanate (FITC) goat anti-rabbit IgG (Boster, Wuhan, China) antibodies, an IL-17 enzyme-linked immunosorbent assay (ELISA) kit (R&D Systems, Minneapolis, MN, USA), phorbol 12-myristate 13-acetate (PMA; Sigma, St.Louis, MO, USA), IL-4 (Sigma), IL-17 (Sigma), oxaliplatin (Sigma), IL-17R Lentivirus shRNA (GenePharma, Shanghai, China), LAMP-2A Lentivirus shRNA (GenePharma, Shanghai, China), and a cyclin D1 overexpression plasmid (GenePharma, Shanghai, China).

### Immunohistochemistry

HCC tissues were fixed in 4% formalin and embedded in paraffin. Sections were incubated with anti-CD68, anti-CD163, or anti-CD206 antibodies and then treated with immunoperoxidase by means of a 3,3′-diaminobenzidine (DAB) kit (Thermo, USA). The sections were examined under a light microscope at 400× magnification. A consensus reading was obtained for discordant cases.

### Immunofluorescence

HCC tissues were incubated with antibodies against CD68 (1:100), CD206 (1:100) or CD163 (1:100) overnight at 4°C. Then, the tissues were washed and incubated with TRITC goat anti-mouse IgG (1:50) or FITC goat anti-rabbit IgG (1:50) secondary antibodies for 1 h at 37°C in the dark. Nuclei were stained with 4′,6-diamidino-2-phenylindole (DAPI) for 10 min. Images were captured with an inverted fluorescence microscope (Olympus, Tokyo, Japan).

### Cell lines and culture conditions

Human SMMC-7721 and HepG2 cell lines were cultured at 37°C in a humidified atmosphere containing 5% CO_2_ in high-glucose Dulbecco's modified Eagle's medium (DMEM) supplemented with 10% heat-inactivated fetal bovine serum (FBS), 100 U/mL penicillin and 100 mg/mL streptomycin. THP1 cells were cultured in RPMI 1640 medium (Gibco, USA) supplemented with 10% heat-inactivated FBS, 100 U/mL penicillin and 100 mg/mL streptomycin at 37°C with 5% CO_2_.

### M2-TAM differentiation and co-incubation with HCC cells

THP-1 cells (2 × 10^5^) were seeded in Transwell permeable (0.4-μm) inserts (Corning, USA) and were treated with 100 ng/mL PMA for 24 h and with 100 ng/mL IL-4 for another 48 h in the upper compartment. The medium was then changed to complete medium. HepG2 cells (2 × 10^5^) or SMMC-7721 cells (2 × 10^5^) were seeded in the lower compartment, followed by 24 h of co-culture. Then, the HepG2 or SMMC-7721 cells were treated with 20 μg/mL oxaliplatin for various times.

### ShRNA knockdown of IL-17R or LAMP-2A and establishment of cell lines

Stable transfectants expressing short hairpin RNAs (shRNAs) against IL-17R (sh-IL-17R) or LAMP-2A (sh-LAMP-2A) were generated through the infection of cells with green fluorescent protein (GFP)-tagged lentiviruses expressing each specific shRNA. A GFP-tagged empty lentivirus (sh-NC) was used as the negative control. Fluorescence microscopy and Western blotting were performed to determine the knockdown efficiency.

### Transfection of the cyclin D1 overexpression plasmid

A Transfection Reagent (Promega, USA) was used to transfect HepG2 cells with a cyclin D1 overexpression plasmid, in accordance with the manufacturer's instructions.

### RNA isolation and quantitative real-time PCR

Total RNA was extracted with an RNeasy kit (Fermentas Life Sciences) in accordance with the manufacturer's protocol. Real-time PCR was performed in 20-μL reactions containing 2 μL cDNA, 0.3 μL of each primer, 7 μL ddH_2_O, 0.4 μL ROX reference dye, and 10 μL fluorescent SYBR Green (TaKaRa Bio, Inc.). Amplification was carried out in 96-well optical plates on a 7300 Real-Time PCR System (Applied Biosystems) at 95°C for 30 s, followed by 45 cycles of 95°C for 5 s and 60°C for 60 s. Each sample was analyzed in triplicate.

### ELISA

IL-17 levels were measured by solid-phase sandwich ELISA (R&D Systems) according to the manufacturer's protocol, and were expressed as pg/mL. Each sample was analyzed in triplicate.

### Western blotting

Cells were lysed in whole-cell lysate buffer containing phenylmethane sulfonyl fluoride and a phosphatase inhibitor. Proteins were blotted by a standard protocol. The primary antibodies included anti-IL-17, anti-IL-17R, anti-BCL-2, anti-BAX, anti-caspase-3, anti-cleaved caspase-3, anti-LAMP-2A, anti-HSC70, anti-cyclin D1, and anti-CDK4. β-actin was used as a loading control. Each experiment was repeated three times, and similar results were obtained.

### Flow cytometry

Cells were harvested, re-suspended and counted. After incubation with Annexin V-FITC (BD Biosciences) and propidium iodide at room temperature in the dark, stained cells were analyzed by flow cytometry (BD Biosciences, San Diego, CA, USA). Data were analyzed by Flowjo Software (Tree Star, Ashland, OR, USA).

### Statistical analysis

Statistical analysis was performed with SPSS 17.0 software. Graphs were constructed in an Image Lab system. Data are expressed as the means ± standard deviations of three independent determinations. Statistical analysis was conducted by either the Student's *t-test* or one-way analysis of variance in comparison with the corresponding controls. Probability values of less than 0.05 were considered to be statistically significant.
